# Bayesian spatio-temporal modeling and prediction of malaria cases in Tanzania mainland (2016-2023): unveiling associations with climate and intervention factors

**DOI:** 10.1186/s12942-025-00408-8

**Published:** 2025-08-01

**Authors:** Lembris Laanyuni Njotto, Wilfred Senyoni, Ottmar Cronie, Anna-Sofie Stensgaard

**Affiliations:** 1 College of Information and Communication Technologies (CoICT), University of Dar Es Salaam (UDSM), Dar Es Salaam, Tanzania; 2 Department of Mathematics and ICT, College of Business Education, Dar Es Salaam, Tanzania; 3 Department of Mathematical Sciences, Chalmers University of Technology & University of Gothenburg, Gothenburg, Sweden; 4 Section for Parasitology and Pathobiology, Department for Veterinary and Animal Sciences, University of Copenhagen, Copenhagen, Denmark

**Keywords:** Malaria cases, Areal data, Standardized Incidence Ratio (SIR), Spatio-temporal model, Random effects, Integrated nested laplace approximations (INLA), Tanzania Mainland

## Abstract

**Background:**

Malaria continues to pose a significant global health challenge, affecting approximately 200 million individuals annually and resulting in an estimated 600,000 deaths each year. In Tanzania, malaria ranks among the top five most commonly reported diseases in healthcare facilities, thus contributing to a substantial burden on the healthcare system. This study analyzed aggregated monthly malaria count data for the period 2016-2023, to explore spatio-temporal trends in malaria risk and assess the effects of climatic factors and vector control interventions across Tanzania mainland regions.

**Methods:**

The Standardized Incidence Ratio (SIR) was used to assess malaria risk distribution, while a Bayesian spatio-temporal model using integrated nested Laplace approximations (INLA) was employed to evaluate the impact of climatic factors and vector control interventions. The model accounted for spatial and temporal effects by using a Conditional Autoregressive (CAR) dependence structure and a random walk of order two (RW2). The analysis was categorized into two age groups, with a cut-off at 5 years.

**Results:**

The study recorded a total of 23.4 million malaria cases in individuals aged 5 years and above, and 17.3 million cases in children under 5 years. The SIR and the model results identified regions with high malaria risk, and the model indicated that from 2016 to 2023, the malaria risk decreased by $11.0\%$ for children under 5 years and by $10.0\%$ for individuals aged at least 5 years. The use of long-lasting insecticide nets (LLINs) reduced the risk of malaria by $1.2\%$ in children under 5 years and by $7.0\%$ in individuals aged 5 years and above. Factors such as minimum temperature, wind speed, and high Normalized Difference Vegetation Index (NDVI) were associated with an increased malaria risk for both age groups. Relative humidity and maximum temperature, both lagged by two months, were associated with an increased malaria risk in children under 5 years, while maximum temperature lagged by one month was associated with increased malaria risk in individuals aged 5 years and above. Similarly, minimum temperature lagged by two and three months was associated with increased malaria risk in individuals aged 5 years and above and in children under 5 years, respectively. In addition, maximum temperature and wind speed lagged by one and three months were associated with decreased malaria risk in both groups.

**Conclusion:**

The environmental factors identified in this study, alongside the spatial mapping, are critical for devising targeted malaria control strategies, especially in regions where LLINs have reduced transmission. These findings are essential for identifying high-risk areas in endemic regions and for prioritizing immediate interventions

## Introduction

Mosquito-borne diseases (MBDs) represent a group of illnesses transmitted to humans through mosquito bites, which involve a range of pathogens, including viruses (arboviruses) and parasitic organisms. Among these, malaria, which is caused by *Plasmodium* parasites transmitted by bites from infected female *Anopheles* mosquitoes, is particularly prominent as a life-threatening disease [1, 2]. Malaria still remains a significant global health threat, with roughly 200 million clinical cases annually; the World Health Organisation (WHO) estimated 249 million cases and 608,000 deaths across 85 countries in 2022 [3]. Africa bears the highest burden of malaria, accounting for $94\%$ of the global cases and $95\%$ of the deaths, with young children being the most vulnerable individuals; they comprise $78\%$ of all malaria deaths in the region [3]. Despite ongoing control efforts, malaria cases have increased in recent years, with Tanzania ranking among the top ten countries in terms of malaria cases and deaths, having contributed about $3.2\%$ of global cases and $4.4\%$ of global deaths in 2022 [3].

Environmental factors play a crucial role in malaria transmission dynamics. Temperature influences the development of the parasite within the vector by affecting the duration of larval development and vector survival [4, 5], while warmer temperatures also increase the feeding frequency of female *Anopheles* mosquitoes [6–8]. Rainfall influences malaria transmission by creating and sustaining mosquito breeding sites, thus boosting vector populations [9, 10]. Although less frequently explored, wind speed can also affect mosquito behavior and malaria transmission. High winds can increase mortality in mosquito larvae due to water turbulence, influence adult mosquito movement (advection), and enhance host-seeking behavior by dispersing ($CO_2$) which attracts mosquitoes to hosts more effectively [11].

Several studies, employing various modeling techniques, have explored the impact of climate variability and climate change on the distribution and intensity of malaria risk in various settings [12–14]. However, these investigations have produced disparate results, potentially stemming from the lack of high-quality malaria data, which is often a consequence of a weak and fragmented nature of national health information systems in many malaria-endemic countries. A further potential reason may be that these modeling approaches vary a great deal in terms of structure and complexity [15]. Establishing an effective monitoring system that can promptly identify malaria cases is crucial for implementing swift and efficient interventions to control or eliminate the disease. Many developing countries, including Tanzania, have adopted the District Health Information Software (DHIS) for this purpose [16]. This system evolved from the Health Management Information System (HMIS), which initially facilitated the reporting of routine health facility data to the Ministry of Health (MoH) through a paper-based reporting and storage system. The transition to the electronic web-based District Health Information Software version 2 (DHIS2) marked a significant upgrade. Moreover, Tanzania’s adoption of the “Test and Treat"campaign has led to an increase in the number of health facility malaria cases confirmed by rapid diagnostic tests (RDTs) [17, 18]. This approach enhances the accuracy of malaria diagnoses and contributes to a more effective response to the disease at the health facility level.

In Tanzania, malaria transmission rates exhibit significant geographical variability, traditionally being more prevalent in low-altitude regions but increasingly reported in high-altitude areas [19]. Transmission follows a distinct seasonal pattern, with major peaks occurring during or after the heavy rains from March to May, and minor peaks following the lighter rains from October to December [20]. This spatial and temporal variability presents a substantial challenge for malaria control, highlighting the need for targeted strategies beyond uniform interventions. To better capture these dynamics, this study first employs the Standardized Incidence Ratio (SIR) to estimate malaria risk across regions and time. By adjusting observed cases for population size, SIR enables more accurate comparisons of relative risk, helping to identify areas and seasons of increased susceptibility. Then, building on this foundation, a Bayesian spatio-temporal model is applied to explore the influence of climatic and intervention-related factors on malaria transmission. These models account for spatial and temporal dependence trends in malaria incidence while incorporating key influential factors [21, 22], offering a refined understanding of how these variables drive malaria dynamics. To ensure a comprehensive assessment, the analysis considers children under five separately from all individuals aged five years and above, acknowledging differences in immunity, exposure patterns, and intervention coverage across these groups. In addition, the predictive performance of the model was evaluated, assessing its ability to reproduce known patterns and forecast future malaria trends. The resulting stratified risk maps and model outputs provides critical insights to support targeted malaria control and elimination efforts, informing evidence-based planning, resource allocation, and the design of effective interventions.

## Materials and methods

### Data sources

#### Tanzania Malaria, interventions and demographic data

This study utilized data from the Tanzania National Health Portal, managed by the Ministry of Health, covering the period from January 2016 to December 2023 [23]. Established in 2015, the portal was designed to centralize access to health information across the country. It aggregates data from multiple sources, including routine health management information system records, surveys, health reports, and publications. Since 2009, Tanzania’s healthcare system has transitioned from paper-based methods to the electronic District Health Information System version 2 (DHIS2). DHIS2 is an open, web-based platform that enables the reporting, analysis, and dissemination of health-related data. It collects data from both private ($26\%$) and public ($74\%$) healthcare facilities and is accessible to authorized healthcare professionals using registered credentials. Most of the DHIS2 data are imported into the Tanzania National Health Portal [23]. Data uploads to the portal occur quarterly after the analysis of data from various reporting sources. These data are freely available from the portal and provide an opportunity to examine both inter- and intra-annual variations in malaria risk within the country, supporting the monitoring and evaluation of malaria programs and contributing to evidence-based decision-making.

The malaria cases data can be accessed within the Tanzania National Health Portal and comprises information on the number of positive Malaria Rapid Diagnostic Tests (mRDTs), positive Malaria Blood Slide (mBS) cases in outpatient departments (OPDs), and clinical malaria cases in OPDs. The malaria cases data are stratified by gender, distinguishing between males and females, and further categorized by age groups, specifically"Under 5 Years"and"5 Years and Above."This stratification is used because the data we have were already structured this way, and we also hypothesize that climate effects differ by age due to variations in physiological vulnerability and immune responses between younger and older individuals. Furthermore, the Tanzania National Health Portal follows a hierarchy of four levels. At the top is the Muhimbili National Hospital, which offers specialized healthcare services. Following this structure are regional hospitals, district hospitals, and health centers at the sector level, all contributing to the Tanzania healthcare system. Tanzania mainland is divided into 26 administrative regions, each with at least one healthcare center. These regions are further subdivided into 184 councils, which act as the primary operational units for government resource allocation and planning of disease prevention and management efforts, with their own budgeting capabilities. Councils are further broken down into wards, which serve as the lower administrative units for resource distribution and disease reporting. This study focuses on examining malaria cases at the regional level.

From the same portal, we obtained demographic data for Tanzania mainland, stratified by regions and further disaggregated by age categories, with the following groups: infants ($<1$ year), young children (1–4 years), children (5–9 years), adolescents (10–14 years), adults of reproductive age (15–49 years), middle-aged individuals (50–60 years), and seniors (60 years and older). This demographic information also includes a gender breakdown. Additionally, we collected data on expectant mothers who received Antenatal Care (ANC) and were concurrently provided with Long-Lasting Insecticidal Nets (LLINs), alongside with the number of children who benefited from LLINs. These nets play a crucial role in malaria prevention, especially among vulnerable groups like pregnant women and young children.

#### Climatic and environmental data

We obtained monthly climate data for each Tanzania mainland region from the NASA Prediction Of Worldwide Energy Resources (POWER), Data Access Viewer (DAV), version 2.4.0 [24]. While the POWER platform serves as an access point, the underlying climate variables are derived from multiple satellite and reanalysis sources. For instance, precipitation data are based on the Global Precipitation Measurement (GPM) Integrated Multi-satellite Retrievals for GPM (IMERG) [25, 26], and temperature, humidity, and wind speed are derived from the Modern-Era Retrospective Analysis for Research and Applications, Version 2 (MERRA-2) ensemble product [27]. The climate data have a spatial resolution of approximately $0.5^\circ \times 0.5^\circ (\approx 50\ km)$ and are available at daily, monthly, and climatological temporal scales. For this study, we extracted monthly averages of the following variables: near-surface air temperature (maximum, minimum, and mean), relative humidity, precipitation, and wind speed. To align the climate data with the administrative boundaries used in our analysis, we aggregated the gridded data spatially to the regional level using a zonal mean approach. Specifically, all grid points within each region geographic polygon were aggregated to produce a single monthly value per variable per region.

For *Normalized Difference Vegetation Index (NDVI)*, we used the Global Information and Early Warning System on Food and Agriculture (GIEWS) [28, 29], as a proxy for moisture and water availability for mosquito breeding and survival. GIEWS utilizes remote sensing data to provide insight on water availability and vegetation health across the globe. NDVI measures the greenness of ground cover and is used as a proxy to indicate the density and"greenness"of vegetation. NDVI values range from $+1$ to $-1$, with high positive values corresponding to dense vegetation (i.e. high availability of water), and low and/or negative NDVI values indicating sparse vegetative cover (indicating low availability of water for vegetation growth).

#### Data cleaning

Since the Tanzania National Health Portal and the DHIS2 database do not differentiate between zero cases and missing values, treating both as blanks, we assumed that in otherwise complete routine monthly malaria reports (from January 2016 to December 2023), any missing values represented true zero cases. In line with WHO recommendations, in this study we focused on two key indicators for malaria diagnosis: the number of malaria-positive Blood Smears (BS) in OPDs and positive mRDTs, as these are considered reliable tools for malaria diagnosis. On the other hand, to ensure the demographic data matched the monthly malaria data, we restructured it into two age groups: children under 5 years (labeled as"< 5 years") and individuals aged 5 years and above ("$\ge 5$ years"). Infants (under 1 year) and young children (1–4 years) were grouped into the "< 5 years" category, while older children (5–9 years), adolescents (10–14 years), adults (15–49 years), middle-aged individuals (50–60 years), and seniors (60+ years) were categorized into the "$\ge 5$ years" group. Furthermore, to facilitate monthly analysis, annual population data for each gender and age group were divided by 12, assuming a consistent population growth rate throughout the year. This approach provided a more accurate representation of population dynamics for model development. Additionally, we carefully calculated the monthly percentage of long-lasting insecticidal net (LLIN) users. This involved summing the number of pregnant women who received LLINs during antenatal care (ANC) visits and the number of children benefiting from LLINs. By dividing this total by the corresponding monthly population, we obtained precise proportions of LLIN users for each month, adding valuable context for our analysis.

### Statistical analysis

Our data encompasses both spatial and temporal information for each data point, making it spatio-temporal in nature. To analyze this spatio-temporal data effectively with stochastic models, it is crucial to account for the spatial locations and corresponding time points associated with the multivariate model’s random variables.

In the realm of spatio-temporal data analysis, different data types can arise depending on how the spatial sampling units are specified within a given area. The data considered in this study were of *areal unit* type, as we analyzed aggregated counts of malaria cases at the regional level (the boundary of each region is known); for other spatial(-temporal) data types, see e.g [21, 30]. This means that we analyze aggregated counts of malaria cases at the regional level, and the boundaries of each region are well defined and known. This approach allows us to gain insights into the multivariate distribution and patterns as well as correlations of malaria, over time, across different regions of Tanzania mainland.

#### Standardized incidence ratio (SIR)

There are different exploratory/non-parametric approaches for assessing the risk of a specific disease for a specific spatial region, which is part of a group of smaller geographic regions that constitute a larger study area. Such approaches allow us to gain insight into how the disease risk varies across different locations and, in turn, can guide subsequent parametric modeling approaches. Moraga [31] addressed this by introducing a method for estimating disease risk in these smaller areas and propose the use of a statistical measure called the Standardized Incidence Ratio (SIR) to quantify disease risk. An SIR value greater than 1 indicates that the disease risk in a given region exceeds what would be expected in a standard population, while a value below 1 suggests the risk is lower than expected.

For each specific region, denoted as area $i=1,\ldots ,n$, at time *t*, the SIR is calculated by comparing the observed number of disease cases ($Y_{it}$) to the expected number of cases in an ideal scenario ($E_{it}$), as expressed by

$$\begin{aligned} SIR_{it} = \frac{Y_{it}}{E_{it}}. \end{aligned}$$

The expected count $E_{it}$ is crucial because it represents the number of disease cases that one would anticipate in region *i* at time *t* if the underlying population would exhibit the same disease incidence as a standard (or regional) population. This comparison allows us to identify regions that deviate from the expected disease risk. To compute the counts $E_{it}$, Moraga [31] suggests a statistical technique known as indirect standardization. This method involves summing up the products of two factors: the disease rate ($r_{jt}^{(s)}$) in various subgroups or strata at time *t*, within the standard population, and the population size of each stratum within region *i* at time *t*. Consequently,

$$\begin{aligned} E_{it} = \sum _{j}r_{jt}^{(s)}n_{jt}^{(i)}. \end{aligned}$$

In some cases, where this information about stratum-specific data is unavailable, we can simply calculate the expected counts using a more straightforward approach, namely

$$\begin{aligned} E_{it} = r_{t}^{(s)}n_{t}^{(i)}, \end{aligned}$$

where $r_{t}^{(s)}$ represents the overall disease rate in the standard population, calculated by dividing the total number of disease cases in the standard population by the total population across all regions. Meanwhile, $n_{t}^{(i)}$ denotes the population of the specific region *i* being analyzed at time *t*. The SIR for each region and year were estimated to assess the spatial variation in malaria risk. This information can help inform public health decisions, resource allocation, and targeted interventions in areas with varying disease risks.

#### Spatio-temporal Bayesian model formulation

While SIRs provide valuable insights into the relative risk of malaria across different regions, they do not address the underlying"why"or"how"questions related to disease risk. To comprehensively understand how various factors or covariates influence malaria risk, a stochastic model becomes essential. Bayesian spatio-temporal models provide a detailed understanding of malaria distribution by estimating risk levels across regions while accounting for spatial and temporal dependencies. A key advantage of this approach is the ability to generate posterior predictive distributions, allowing for robust predictions while incorporating uncertainty in model parameters. Additionally, Bayesian models can integrate prior information, improving estimate stability, particularly when data is sparse or incomplete. Ultimately, this enhances the accuracy and reliability of conclusions regarding disease distribution and its risk factors.

The number of malaria cases, denoted by $y_{it}$, is observed across 26 regions ($1 = 1,\cdots , 26$) over 96 time points (*t*, representing the months from 2016 to 2023). To model this data, we define $Y_{it}$ as the random variable representing the malaria case counts. Given that malaria cases are count data, we assume $Y_{it}$ follows a Poisson distribution, which is a common and appropriate choice for modeling such data. In the employed model, the mean is given by $E_{it}\theta _{it}$, where $E_{it}$ represents the expected counts of the malaria cases, and $\theta _{it}$ signifies the relative risk associated with region *i* at time *t*. The relative risk, $\theta _{i}$, quantifies whether region *i* has higher $(\theta _{i}> 1)$ or lower $(\theta _{i}<1)$ risk than the average risk in the standard population. Additionally, covariates are often included to quantify specific risk factors, while other random effects are incorporated to address further sources of variability.

This statistical model can be expressed as

1 $$\begin{aligned} Y_{it}&\sim \textrm{Poisson}\left( \lambda _{it}\right) , \nonumber \\ \lambda _{it} = \log {\left( \theta _{it}\right) }&= \alpha + u_i + v_i + T_t + \varvec{X}\varvec{\beta } + \log {(E_{it})}, \end{aligned}$$

where the log relative risk can be obtained as

2 $$\begin{aligned} \log {\left( \frac{\theta _{it}}{E_{it}}\right) }&= \alpha + u_i + v_i + T_t + \varvec{X}\varvec{\beta }, \nonumber \\ \alpha&\sim N\left( 0, \tau _0^{-1}\right) , \nonumber \\ u_i|u_{-i}&\sim \ N\left( {\bar{\mu }}_{\delta _i},\frac{{\sigma _u}^2}{n_{\delta _i}}\right) , \nonumber \\ v_i&\sim N\left( 0,\sigma _v^2\right) . \end{aligned}$$

The logarithm of the relative risk, $\theta _{it}$, is modeled as the sum of several components, including spatial and temporal structures that account for both spatial and spatio-temporal correlations. The expected number of cases for each region over time, $\log (E_{it})$, is included as an offset to adjust for varying exposure levels. This offset acts as a correction factor and is assumed to have a fixed regression coefficient of 1 [21]. Here, $\alpha$ is the intercept, quantifying the overall or average risk for all regions, essentially the baseline level of risk, $u_i$ is the spatially structured region-specific random effect, which accounts for spatial correlation among regions. It allows for smoothing among adjacent regions, considering that regions closer to each other tend to have more similar risk levels. $v_i$ is the unstructured residual, modeled using exchangeability among the 26 regions. It captures the additional heterogeneity in the counts of malaria cases attributed to unobserved risk factors that are not spatially structured. In other words, it accounts for unexplained variation and random noise. $T_t$ denotes the temporal effects, capturing how the risk of malaria cases changes over time. This can be defined as either a parametric or a nonparametric structure, depending on the specific temporal patterns we want to capture. $\varvec{X}$ is a matrix of covariates (fixed effects) for each region-time combination, and $\varvec{\beta }$ is a matrix of coefficients corresponding to these covariates. It allows the inclusion of additional explanatory variables in our model, explaining how they affect the log of the expected risk.

The notation $u_i|u_{-i}$ refers to the conditional distribution of the random effect $u_i$ (associated with a specific region *i*) given all other random effects $u_{-i}$, where $u_{-i}$ represents the collection of random effects for all regions except region *i*. This formulation suggests that we are dealing with a multivariate random vector (Markov random field (MRF)) where the dependence between regions is spatially structured. In this case, the spatial dependence is modeled through a Conditional Autoregressive (CAR) model, which is a specific type of MRF. In a CAR model, the random effect for a particular region depends only on its neighboring regions rather than on all other regions globally, which is a characteristic of MRFs. The conditional distribution $u_i|u_{-i}$ is typically assumed to follow a normal distribution with a mean that is a function of the random effects of the neighboring regions and a variance inversely related to the number of neighbors.

In the following subsections, we outline several adaptations to the model in equation (1), where the temporal component can be modeled using either parametric or semi-parametric approaches.

##### A parametric version of the model

Bernardinelli et al. [32] proposed a spatio-temporal model with parametric time trends that expresses the logarithm of the relative risks in equation (1), as

3 $$\begin{aligned} \log {\left( \theta _{it}\right) } = \alpha + u_i + v_i + (\beta + \delta _i)t + \varvec{X}\varvec{\beta } + \log {(E_{it})}. \end{aligned}$$

Here, $\alpha$ is the intercept, $u_i + v_i$ is an area random effect, $\beta$ is a global linear trend effect, and $\delta _i$ is an interaction between space and time representing the difference between the global trend $\beta$ and the region specific trend. The components $u_i$ and $\delta _i$ are modeled using a conditional autoregressive (CAR) model, while $v_i$ are independent and identically distributed normal variables. This modeling approach allows each region to have its own unique time trend, characterized by a spatial intercept represented by $\alpha + u_i + v_i$ and a slope determined by $\beta + \delta _i$.

To ensure identifiability of the resulting model, a constraint is applied to the variables $\varvec{\delta } = \{\delta _1, \cdots ,\delta _2, \delta _n\}$ and $\textbf{u} = \{u_1, u_2,\cdots , u_n\}$, requiring that their sum equals zero. This constraint is crucial for identifiability because it removes the redundancy in the model parameters, ensuring that the effects attributed to these variables can be uniquely determined. Without such a constraint, the model would have an infinite number of solutions because adding a constant to all $\delta _i$ or $u_i$ values would not change the model’s fit to the data. By constraining the sum to zero, we effectively ensure that the parameters are uniquely determined. The value $\delta _i$ is referred to as the"differential trend"and it is signifying the extent to which the time trend in region *i* differs from the overall time trend $\beta$. That is, when $\delta _i < 0$ it indicates that the area-specific trend is less steep than the average trend. In other words, the rate of change in that particular area is lower compared to the overall trend. Conversely, when $\delta _i > 0$ it suggests that the area-specific trend is steeper than the average trend, indicating a faster rate of change in that specific area compared to the overall trend.

##### A semi-parametric version of the model

The parametric model described in equation (3) assumes a log linear relationship between the disease risk and time, but this may not fully capture the complex dynamics of how disease risk evolves over time across different regions. To address this limitation, Knorr-Held [33] proposed an enhanced model that incorporates both spatial and temporal random effects, as well as an interaction between space and time:

4 $$\begin{aligned} \log {\left( \theta _{it}\right) } = \alpha +u_i + v_i + \gamma _t + \phi _t +\delta _{it} + \varvec{X}\varvec{\beta } + \log {(E_{it})}. \end{aligned}$$

In this model, $\alpha$ represents the intercept, while $u_i+v_i$ denotes the spatial random effects. These are parameterized in the same way as in Equation (1), with $u_i$ following the CAR model and $v_i$ being independent and identically normally distributed random variables. The terms $\gamma _t$ and $\phi _t$ represent temporal random effects which allow the model to capture complex temporal dynamics that a simple linear trend might miss. $\gamma _t$ is used to account for broad temporal trends and patterns, such as seasonality or long-term shifts in disease risk. It is modeled as a random walk, which can either be of first-order (RW1),

$$\begin{aligned} \gamma _t|\gamma _{t-1} \sim N(\gamma _{t-1},\sigma _{\gamma }^2), \end{aligned}$$

or of second order (RW2),

$$\begin{aligned} \gamma _t|\gamma _{t-1}, \gamma _{t-2} \sim N(2\gamma _{t-1} - \gamma _{t-2},\sigma _{\gamma }^2). \end{aligned}$$

In a second-order random walk (RW2), the parametrization $2\gamma _{t-1} - \gamma _{t-2}$ helps capture more complex changes over time by looking at data from two previous time points. This approach allows the model to understand not just how the trend is changing at the moment, but also how the rate of change itself is shifting. This makes the model better at reflecting more intricate patterns in the data, such as speeding up or slowing down trends, rather than just assuming a steady, linear change. Furthermore, $\phi _t$ represents an unstructured temporal effect that captures additional variability not explained by $\gamma _t$. This effect is modeled as an independent and identically distributed normal variable:

$$\phi _t \sim N(0, \sigma _{\phi }^2).$$

To capture the interaction between space and time, the model includes the parameter $\delta _{it}$ which can be specified in different ways by combining the structures of the random effects which are interacting. This parameter accounts for the differences in the time trend (of malaria cases) across various regions, enabling an examination of how the time trend varies geographically. Knorr-Held et al. [21, 33] proposed four different types of interactions, namely, interaction between the effects $(u_i, \gamma _t),(u_i,\phi _t),(v_i,\gamma _t)$ and $(v_i,\phi _t)$. These interaction are summarized in Table 1.

**Table 1:** Interaction Types and Structure Matrices

Interaction Type	Structure matrix $\textbf{R}_\delta$	Parameters interacting	Rank
I	$\textbf{R}_\delta =\textbf{R}_u\otimes \textbf{R}_\phi =\textbf{I}\otimes \textbf{I}=\textbf{I}$	$u_i$ and $\phi _t$	*nT*
II	$\textbf{R}_\delta =\textbf{R}_u\otimes \textbf{R}_\gamma$	$u_i$ and $\gamma _t$	$n(T-2)$ for RW2
III	$\textbf{R}_\delta =\textbf{R}_\phi \otimes \textbf{R}_v$	$v_i$ and $\phi _t$	$(n-1)T$
IV	$\textbf{R}_\delta =\textbf{R}_v\otimes \textbf{R}_\gamma$	$v_i$ and $\gamma _t$	$(n-1)(T-2)$ for RW2

Table 1 presents different interaction models, each with its own set of assumptions and characteristics. In the Type I interaction model, it is assumed that two unstructured effects, denoted by $u_i$ and $\phi _t$, interact with each other. These effects are considered to have no inherent spatial or temporal structure. Additionally, there is no spatial or temporal structure attributed to their interaction. Essentially, this model assumes no specific patterns or relationships among these effects and the matrix $\textbf{R}_\delta$ (describes the way different random effects or parameters interact with each other) has a rank *nT* (provides insights into the dimensions and complexity of the interactions being modeled). In the Type II interaction model, a structured temporal main effect, $\gamma _t$, is combined with an unstructured spatial effect $u_i$. In this scenario, the spatial effect is not subject to any specific constraints ($\textbf{R}_u = \textbf{I}$), while the temporal effect is structured based on a specified neighborhood structure, often established through a first or second-order random walk ($\textbf{R}_\gamma$). This results in an assumption that, for each area, the parameter $\{\delta _{i1}, \cdots , \delta _{iT}\}$ exhibits an autoregressive structure over time. This structure is independent of other areas, and the matrix $\textbf{R}_\delta$ has a rank determined by the choice of random walk, either $n(T - 1)$ for first-order or $n(T - 2)$ for second-order. The Type III interaction model combines an unstructured temporal effect, $\phi _t$, with a spatially structured main effect, $v_i$. In this case, there is no specific temporal structure associated with ($\textbf{R}_\phi = \textbf{I}$), while the spatial effect is structured using the Conditional Autoregressive (CAR) specification ($\textbf{R}_v$). This leads to the assumption that the parameters for each time point, ${\delta _1,\cdots ,\delta _n}$, possess a spatial structure that is independent of other time points. The matrix $\textbf{R}_\delta$ has a rank of $T(n - 1)$. The Type IV interaction model assumes that both the spatially and temporally structured effects interact. This implies that the temporal dependence structure for each area is not solely independent but depends on the temporal patterns of neighboring areas as well. The structure matrix in this model is formed as the Kronecker product, and its rank depends on the order of the random walk chosen, either $(n - 1)(T - 1)$ for a random walk of order 1 or $(N - 1)(T - 2)$ for a random walk of order 2.

#### Model fitting and validation

##### Model fitting

Given that our response variable represents counts, as we have already indicate, the natural model to use would typically be the Poisson model. However, the Poisson model assumes that the mean and variance are equal, which is often not the case in real-world scenarios. We may often encounter overdispersion [34, 35], where the variance exceeds the mean, or there might be an excessive number of zeros in the data [36]. In cases of overdispersion, the Poisson model may not be suitable, and alternative models that can accommodate a larger variance relative to the mean become necessary. One such model is the negative binomial, where each $Y_{it}$ follows a negative binomial distribution. This model is recommended as it introduces an additional parameter to independently model the mean and variance, effectively handling overdispersion [34]. Furthermore, when the dataset exhibits an excessive number of zeros, such as in regions with low malaria transmission or specific seasons, zero-inflated models are valuable [36]. Such models address the surplus of zeros by incorporating additional parameters to capture structural and sampling issues.

During the model selection process, we utilized the Watanabe-Akaike Information Criterion (WAIC), Marginal log likelihood (mlik), and Deviance Information Criterion (DIC). These metrics offer unique valuable insights into the goodness of fit and model complexity. The WAIC and DIC allows us to assess the balance between these two factors, with smaller values indicating better fitting models that offer a favorable trade-off between accuracy and complexity. The Marginal log likelihood provides an estimation of the average likelihood of the observed data given the model. It serves as a measure of how well the model captures the observed data, with higher values indicating better fit. The DIC is a generalization of the Akaike Information Criterion (AIC), explicitly for Bayesian model comparison, with smaller values indicate better fitting models.

Given that climate variables are often highly correlated, there is a potential risk of multicollinearity when fitting a model. Multicollinearity occurs when two or more predictor variables are highly correlated, meaning they offer similar information [37]. This can lead to unreliable estimates of coefficients, making it difficult to assess the individual effect of each predictor. To assess and address multicollinearity, we specifically used the Variance Inflation Factor (VIF) [37], which quantifies how much the variance of a regression coefficient is inflated due to collinearity with other predictors. A VIF value exceeding 10 is typically considered indicative of significant multicollinearity, warranting further investigation or remedial action. This method helps in reducing the impact of correlated predictors on the model, ensuring more reliable and interpretable results.

##### Model validation

Before conducting exploratory analysis and model fitting, we divided the dataset into training and test sets. The training set, encompassing the first 84 months (January 2016 to December 2022), was used for training the model. The test set, covering the remaining 12 months (January to December 2023), was used for testing the performance of the model on unseen data. We evaluated the predictive performance of the optimal model by comparing observed malaria cases with predicted cases from the training set and forecasted cases from the test set, assessing how accurately the model captured the actual malaria trends. In addition to directly comparing predicted and observed cases, we used a Receiver Operating Characteristic (ROC) curve to assess the model’s ability to distinguish between high-risk and low-risk malaria areas. To construct the ROC curve, we set a threshold of 1 to categorize the SIR values. SIR values greater than 1 were labeled as 1, indicating a predicted high malaria risk, while values equal to or less than 1 were labeled as 0, indicating low or no malaria risk. The ROC curve plots the true positive rate (sensitivity) against the false positive rate (1-specificity) at various threshold levels, providing a visual measure of the model’s discrimination ability. Moreover, we quantified the model’s performance by calculating the Area Under the Curve (AUC). The AUC gives a single numeric value summarizing the ROC curve, where 0.5 indicates performance equivalent to random guessing, and a value closer to 1 suggests strong predictive accuracy. This approach allowed us to robustly evaluate how well the model distinguished between high-risk and low-risk malaria areas, providing key insights into its practical utility for malaria prediction.

All data management and statistical analysis was performed in the software R, version 4.4.2 [38]. The Bayesian hierarchical model was estimated using Integrated Nested Laplace Approximation (INLA) package (R-INLA). INLA is a deterministic algorithm specifically developed for Bayesian inference in latent Gaussian and spatial models [39, 40]. It is a robust estimation method that combines analytical approximation and numerical integration to derive the approximate posterior distribution of parameters [21, 41]. Compared to the traditional Markov Chain Monte Carlo (MCMC) methods, Bayesian estimation using INLA offers significant computational advantages, allowing for faster estimation times [21].

## Results

### Descriptive analysis

Between January 2016 and December 2023, a total of 40.7 million malaria cases were recorded across all Tanzania mainland regions, with an average of 1.5 million cases per region. The highest number of cases during this period was 6.6 million cases, in 2019, while the lowest was 3.4 million cases, in 2022.

**Fig. 1:**
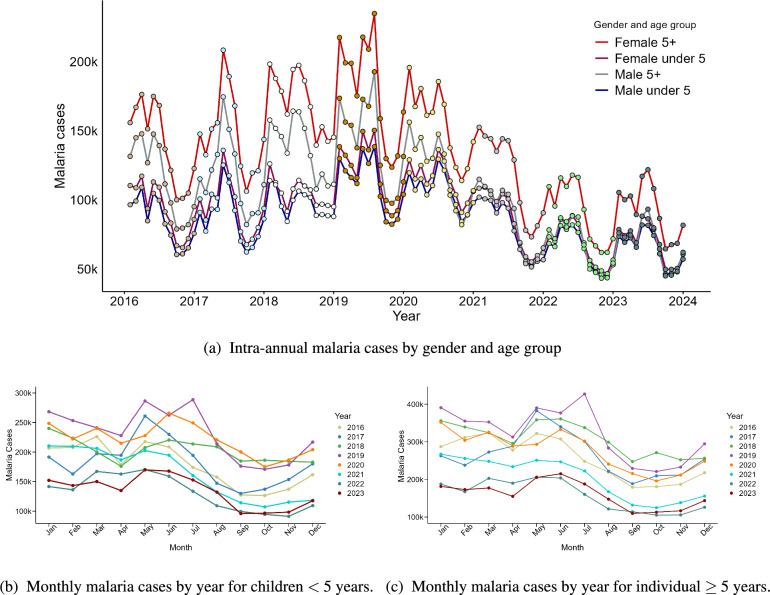
Intra- and inter-annual malaria cases by age and gender in all Tanzania mainland regions from 2016 to 2023

Figure 1 depicts the variation in malaria cases in Tanzania mainland from January 2016 to December 2023, showing significant fluctuations in transmission across different years, age groups, and genders. The lines representing different age groups reveal that individuals aged five years and above had a higher number of malaria cases compared to those under five years of age. However, this should be viewed in the context of the larger population size in the older age group. While the number of malaria cases is higher in the older age group due to its larger population size, the risk or likelihood of contracting malaria may actually be higher for children under five years. This means a larger proportion of children in the younger age group are likely to be more affected by malaria, despite the lower number of reported cases. Furthermore, the same plot illustrates that females had a somewhat higher number of malaria cases compared to males across all age groups. This observation could be attributed by the higher number of females in the country and the fact that women tend to visit health centers more frequently, either for treatment or pregnancy-related care.

The intensity and timing of the seasonal peak of malaria cases vary from year to year for each age group (Figure 1b and 1c). For both groups, malaria cases typically peak from December to July, coinciding with the high rainfall season from November to May and relatively warm conditions. This peak is followed by a decrease in cases from August to October, corresponding to a period of low or no rainfall, suggesting fewer malaria cases. However, this trend reverses with an increase in cases in November, aligning with the moderate rainy season in October and November in some regions of the country. It is notable that monthly malaria cases remained consistently high in 2018, 2019, and 2020 for both age groups, compared to other years.

#### Spatial and temporal variations in malaria SIR

**Fig. 2:**
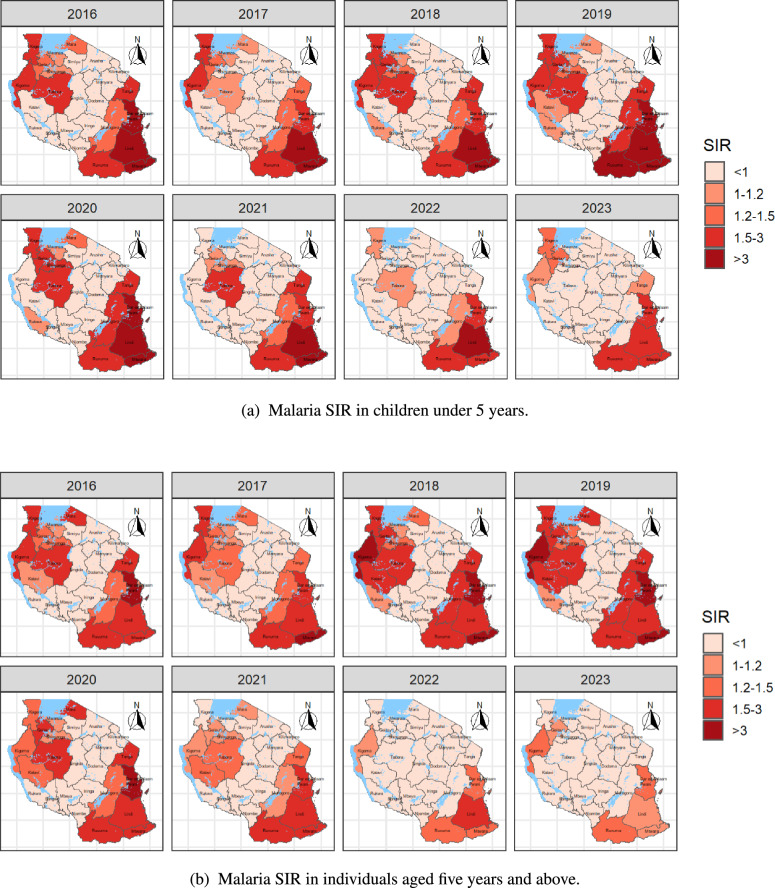
Spatial and temporal distribution of malaria Standardized Incidence Ratio (SIR) by region in Tanzania Mainland from 2016 to 2023 for children under 5 years and for individuals aged at least 5 years. Regions highlighted in red indicated an SIR greater than 1, indicating a somewhat higher malaria risk, while regions with an SIR of 1 or less indicate a relatively lower malaria risk

Figure 2 depicts the spatial and temporal variations in malaria SIR for children under five years of age (Figure 2a) and for individuals aged five years and above (Figure 2b) across Tanzania mainland regions from 2016 to 2023. The regions shaded in shades of red, from light to dark, represent areas with ($SIR > 1$), i.e. those regions with a higher malaria risk compared to the standard population. The maps reveal a consistent pattern of malaria cases across various regions in both age groups. In both groups, the Eastern Zone (which includes Tanga, Dar es Salaam, and Pwani), the Southern Zone (comprising Lindi, Mtwara, and Ruvuma), and the Lake Zone (including Kagera, Kigoma, and Tabora) consistently exhibit a higher malaria burden compared to other regions throughout the study period. Notably, the risk is somewhat higher in children under five years of age in the regions of Lindi and Mtwara. Some regions experience a somewhat lower risk of malaria for both age groups. These include the Northern Zone with the Arusha, Kilimanjaro, and Manyara regions; the Southern Highlands Zone with regions such as Mbeya, Njombe, and Songwe; and the Central Zone, encompassing the Dodoma, Singida, and Iringa regions. Moreover, the maps provide valuable insights into the spatial and temporal correlations in the malaria risk. This becomes evident by the clustering of similar colors in neighboring regions over time, indicating that geographically proximate regions tend to exhibit similar patterns of malaria cases, both in their spatial distribution and temporal changes. The global spatial autocorrelation of malaria cases was evaluated using Moran’s I statistic, calculated based on the total number of cases aggregated by region over the study period. The results revealed a Moran’s I value of $0.41399\ (p = 0.0014)$ for children under five years of age and $0.36542\ (p = 0.002)$ for individuals aged five years and above. These statistically significant values indicate a clear spatial clustering of malaria burden in both age groups. Regions with high case counts tend to be adjacent to other high-burden areas, while regions with lower cases are similarly clustered together. This pattern highlights the geographical concentration of malaria cases, suggesting the potential influence of localized environmental, climatic, and intervention-related factors.

#### Relationship between malaria SIR and climatic factors

During the analysis, a high correlation was observed between average temperature and both maximum and minimum temperatures, raising concerns about multicollinearity, which could compromise model accuracy and interpretation. To address this, a variance inflation factor (VIF) analysis was conducted, identifying average temperature as the primary contributor to multicollinearity. As a result, maximum and minimum temperatures were retained in the model, as they demonstrated lower VIF values.

**Fig. 3:**
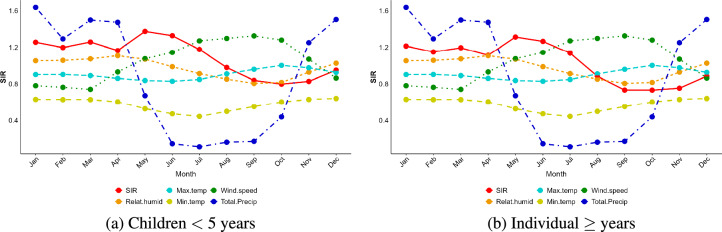
Relationship between standardized malaria cases (SIR) and time-series plots of scaled weather variables for: (**a**) children under five years of age and (**b**) individual aged five years and above.

The relationships between the Standardized Incidence Ratio (SIR) and various climatic variables at different scales throughout the year, for both age groups, are visualized in Fig. 3. In this figure we observe that the pattern of SIR closely aligns with precipitation trends, showing higher values during the rainy season (December to June) and lower values during the drier months (July to October). Regarding temperature, SIR decreases slightly during periods of high maximum temperature, particularly between July and November. Conversely, SIR exhibits a weak positive association with minimum temperature, with slightly higher values during the warmer months (November to April). Additionally, SIR is positively correlated with relative humidity during the wet season (November to May), reflecting higher disease incidence in more humid conditions. In contrast, SIR shows an inverse relationship with wind speed, as periods of lower wind speeds during the rainy season coincide with increased SIR values.

Table 2 presents Pearson correlation coefficients between monthly malaria cases and climatic factors (precipitation, maximum and minimum temperature, relative humidity, and wind speed) across lags of 0 to 3 months for both age groups. Precipitation yielded a significant correlation for children under five years of age up to lag 1, implying that rainfall from the previous month was associated to malaria risk in children. For individuals aged five years and above, the correlation extended up to lag 2, indicating that rainfall had a more prolonged effect on older individuals, potentially due to a longer incubation period or different exposure dynamics. Maximum temperature showed significant positive correlations across all lags for children under five years of age, indicating that higher temperatures in the past 1 to 3 months are consistently associated with an increased risk of malaria. For individuals aged five years and above, the effect was significant only up to lag 2, suggesting a shorter-term effect of temperature on malaria risk in older individuals. Minimum temperature showed significant positive correlations at all lags for all age groups, indicating that minimum temperature in the past 1 to 3 months can contribute to a higher malaria risk, regardless of age. Relative humidity had significant positive correlations at all lags for individuals aged at least five years, meaning that higher humidity in previous months increased malaria risk in this group. For children under five years, the relationship with relative humidity was only significant up to lag 1, suggesting a shorter-term impact on younger children. Wind speed showed consistent negative correlations across all lags and age groups, meaning that higher wind speeds were associated with a decreased malaria risk. This effect was consistent over time, regardless of the age group, suggesting that wind may play an important role in reducing mosquito transmission, possibly by disrupting mosquito flight patterns or dispersing larvae.

**Table 2:** Pearson correlation between monthly malaria incidence and climatic variables analyzed at four lag intervals: Lag 0 (current month), Lag 1 (previous month), Lag 2 (two months prior), and Lag 3 (three months prior)

Climatic factor	Children $< 5$ years				Individuals $\ge 5$ years			
	Lag 0	Lag 1	Lag 2	Lag 3	Lag 0	Lag 1	Lag 2	Lag 3
Precipitation	0.0648 $^{\text {a}}$	0.0663 $^{\text {a}}$	0.0419	0.0217	0.0873$^{\text {a}}$	0.0756$^{\text {a}}$	0.0627$^{\text {a}}$	0.0325
Max. temperature	0.1075$^{\text {a}}$	0.129$^{\text {a}}$	0.1079$^{\text {a}}$	0.0655$^{\text {a}}$	0.0955$^{\text {a}}$	0.1304$^{\text {a}}$	0.0849$^{\text {a}}$	0.0093
Min. temperature	0.2390$^{\text {a}}$	0.2191$^{\text {a}}$	0.1642$^{\text {a}}$	0.0984$^{\text {a}}$	0.3348$^{\text {a}}$	0.3141$^{\text {a}}$	0.2245$^{\text {a}}$	0.1159$^{\text {a}}$
Relative humidity	0.1116$^{\text {a}}$	0.0676$^{\text {a}}$	0.0416	0.0376	0.1594$^{\text {a}}$	0.10177$^{\text {a}}$	0.0821$^{\text {a}}$	0.0898$^{\text {a}}$
Wind speed	−0.1630$^{\text {a}}$	−0.1665$^{\text {a}}$	−0.1681$^{\text {a}}$	−0.1654$^{\text {a}}$	−0.1299$^{\text {a}}$	−0.1310$^{\text {a}}$	−0.1536$^{\text {a}}$	−0.1612$^{\text {a}}$

$^{a}$ Statistically significant correlations at the $5\%$ level

### Model fit

We compared three models (in terms of distribution for the response variable): the Poisson, Negative Binomial, and the Zero-Inflated Negative Binomial. The results (Table 3) indicated that both the Deviance Information Criterion (DIC) and Watanabe-Akaike Information Criterion (WAIC) were lower for the Negative Binomial model compared to the Poisson model, while the marginal likelihood (mlik) was higher. These findings suggest that the Negative Binomial model provided a better fit for the data, both for children under five years of age and for individuals aged five years and above. When we examined the Zero-Inflated Negative Binomial model, there was no significant improvement in these metrics compared to the Negative Binomial model, indicating that the Negative Binomial model was the most suitable and effective choice for capturing the malaria case dynamics in our dataset.

We also assessed the contribution of climatic factors and the proportion of LLIN usage to malaria cases by comparing seven distinct spatio-temporal Negative Binomial models. These models were structured as follows: the first was a non-spatial model, the second incorporated a spatial component, the third included both spatial and temporal components, and the fourth through seventh models accounted for various interactions between time and regions. Based on the DIC, WAIC, and mlik, we found that the spatio-temporal Negative Binomial model with type I interaction achieved the smallest DIC and WAIC, and the largest mlik, demonstrating the best fit among all models (see Table 3).

**Table 3:** Model selection and comparisons: we first choose the model distribution by comparing three distribution (Poisson, Negative Binomial and Zero inflated Negative Binomial), then we compare several model using the chosen distribution

Model	Children $< 5$ years			Individual aged $\ge 5$ years		
	DIC	WAIC	Mlik	DIC	WAIC	Mlik
Poisson	8814647.07	370781.23	−6446288.33	11351175.49	415013.54	−6763198.11
Negative Binomial	**48108.63**	**48106.43**	**−24199.19**	**49603.28**	**49601.08**	**−24964.50**
Zero inflated Negative Binomial	48108.79	48109.18	−24203.31	49606.11	49603.57	−24970.99
Choosing the best negative binomial spatio-temporal model						
Non-spatial model	48109.63	48108.43	−24199.19	49603.28	49601.09	−24964.50
Spatial model	42395.33	42404.42	−21436.57	44861.55	44866.83	−22681.99
Spatio - temporal model	42031.46	42042.47	−21312.20	44469.51	44474.57	−22508.91
Spatio - temporal model with type I interaction	**33549.56**	**34843.39**	**−21292.36**	**44030.54**	**44044.00**	**−22655.79**
Spatio - temporal model with type II interaction	41817.66	41836.99	−21399.90	44045.86	44059.39	−22524.67
Spatio - temporal model with type III interaction	490719.25	41987.43	−21341.20	44202.28	44223.46	−22452.30
Spatio - temporal model with type IV interaction	41806.96	41828.07	−21530.54	44038.65	44073.36	−22467.69

The bold values highlights the model that outperforms the others, having lower DIC and WAIC, as well as a higher mlik

#### Model validation

**Fig. 4:**
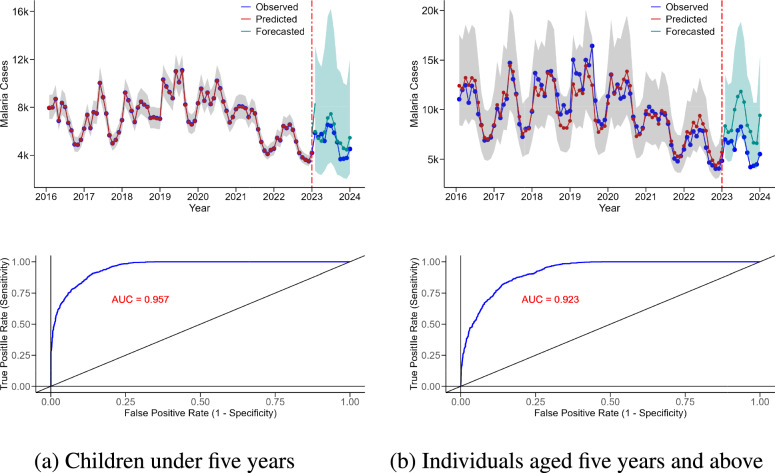
Model validation for malaria cases in two age groups. The plots display observed malaria cases in blue, predicted cases (in the training set) in red, and forecasted cases in cyan for the year 2023. The model was fitted using data from 2016 to 2022 and validated against the 2023 cases data. The shaded region represents the $95\%$ credible interval (CI) for the predictions. The area under the curve (AUC) values for the Receiver Operating Characteristic (ROC) curves are provided at the bottom, further indicating the predictive performance of the model.

The relationship between the predicted and forecasted malaria cases versus the observed cases is illustrated in Figure 4 for both age groups. The observed malaria cases (blue) closely match the predicted values (red) during the training period (2016-2022), demonstrating the models ability to capture underlying trends and seasonal patterns. Furthermore, the forecasted values (cyan) align well with the observed data for the year 2023, indicating that the models generalize effectively to future unseen data. This alignment highlights the model accuracy in fitting historical data and proficiency in forecasting future trends. The model capability to identify peaks and troughs in malaria case patterns further signifies its sensitivity to variations in malaria risk, allowing it to reliably pinpoint periods of high and low malaria cases.

We further evaluated the model’s ability to predict malaria cases using the Receiver Operating Characteristic (ROC) curve and Area Under the Curve (AUC). As shown in the lower panel of Figure 4, the ROC curve approached the upper-left corner for both age groups, indicating the model’s strong capacity to distinguish between high and low/no malaria risk scenarios. To quantify this performance, we calculated the AUC based on the band scores, yielding a value of 0.957 for children under five years of age and 0.923 for individuals aged five years and above. These results confirm the model’s high predictive accuracy.

#### Effects of climatic factors and intervention on spatio-temporal changes in malaria cases

The estimated relative risks (RR) of the malaria cases, determined using the Bayesian Negative Binomial spatio-temporal model with type I interaction, for the years 2016–2023, are shown in Table 4. For children under 5 years of age, the RR was 0.89, indicating a $11.0\%$ decreased risk of malaria for each unit increase in year from 2016 to 2023. Meanwhile, for individuals aged 5 years and above, the RR was 0.90, reflecting a $10.0\%$ decreased risk of malaria for each unit increase in a year over the same period. The use of long-lasting insecticide nets (LLIN) showed a significant protective effect for both age groups. A $1\%$ increase in the proportion of households distributed with at least one LLIN was associated with a $1.2\%$ decreased risk of malaria for children under 5 years of age and a $7.0\%$ decreased risk of malaria for individual aged at least 5 years.

Climatic variables revealed distinct impacts on the malaria risk. Precipitation showed a non significant effect on the malaria risk for either age group. Relative humidity in the current month showed a non-significant increase in malaria risk, while a two-month lag in relative humidity significantly increased malaria risk in children under 5 years of age. The current month’s maximum temperature was significantly associated with a decreased risk of malaria for both age groups, while minimum temperature showed a positive association with increased malaria risk. Specifically, for each 1$^\circ$C increase in maximum temperature, malaria risk decreased by $3.1\%$ for children under 5 years and $2.8\%$ for individuals aged 5 years and above. However, the lagged effects of maximum temperature revealed slightly, yet statistically significant, increases in malaria risk. A one-month lag was associated with a $0.6\%$ increased malaria risk for individuals aged 5 years and above, while a two-month lag led to a $0.3\%$ increased risk of malaria for children under 5 years of age. Conversely, for each 1$^\circ$C increase in minimum temperature during the current month, the risk of malaria increased by $4.4\%$ for children under 5 years of age and $3.9\%$ for individuals aged 5 years and above. Lagged effects of minimum temperature also indicated an increased risk of malaria: a two-month lag resulted in a $1.2\%$ increased risk for individuals aged 5 years and above, while a three-month lag corresponded to a $0.5\%$ increased risk for children under 5 years.

For each unit increase in wind speed during the current month, the risk of malaria increased by $12.0\%$ for children under 5 years of age and $7.7\%$ for individuals aged 5 years and above. However, at a one-month and three-month lag, wind speed significantly reduced the risk of malaria for both age groups. Areas with dense vegetation (NDVI > 0.5) were associated with a substantially high risk of malaria compared to areas with sparse or no vegetation. The risk of malaria increased by $25.8\%$ for children under 5 years of age and $24.2\%$ for individuals aged 5 years and above in regions with dense vegetation.

**Table 4:** Estimated relative risks (RR) and $95\%$ credible intervals (CI) of malaria cases from Bayesian negative binomial Spatio-temporal model with type I interaction, including climatic and interventions effects, and the random effects.

Variables	Children $<5$ years	Individuals $\ge 5$ years
	RR (95%CI)	RR (95%CI)
Fixed effects		
Intercept	0.545 (0.485, 0.613)*	0.623 (0.556, 0.698)*
Year	0.890 (0.874, 0.906)*	0.90 (0.880, 0.912)*
Proportion usage of LLIN	0.988 (0.986, 0.989)*	0.930 (0.925, 0.936)*
Climatic variables		
Precipitation	0.996 (0.986, 1.005)	0.9995 (0.991, 1.008)
Relative humidity	1.00005 (0.998, 1.002)	1.001 (0.999, 1.003)
Relative humidity lag 2	1.002 (1.0008, 1.0029)*	
Maximum temperature	0.969 (0.962, 0.977)*	0.972 (0.965, 0.979)*
Maximum temperature lag 1		1.006 (1.003, 1.009)*
Maximum temperature lag 2	1.003 (1.0013, 1.0050)*	
Minimum temperature	1.044 (1.031, 1.057)*	1.039 (1.027, 1.051)*
Minimum temperature lag 2		1.012 (1.009, 1.015)*
Minimum temperature lag 3	1.005 (1.0007, 1.0093)*	
Wind speed	1.120 (1.079, 1.162)*	1.077 (1.042, 1.114)*
Wind speed lag 1	0.921 (0.896, 0.947)*	0.950 (0.919, 0.981)*
Wind speed lag 3	0.941 (0.919, 0.964)*	0.962 (0.947, 0.976)*
NDVI ($\le 0.3$)	1	1
0.31 - 0.5	1.115 (0.990, 1.253)	1.094 (0.983, 1.219)
$> 0.5$	1.258 (1.1007, 1.437)*	1.242 (1.098, 1.405)*
Random effect		
nbinomial observations (1/overdispersion)	374.72 (68.32, 1558.33)	11.69 (9.10, 14.54)
Precision for structured spatial component ($u_i$)	0.19 (0.10, 0.31)	0.36 (0.19, 0.61)
Precision for unstructured spatial component ($v_i$)	1648.06 (806.93, 3033.01)	1292.01 (680.25, 2302.68)
Precision for temporally unstructured random effects ($\phi _j$)	1925.32 (193.04, 9164.18)	5250.87 (241.89, 28699.24)
Precision for temporal structure random effects ($\gamma _j$)	263.22 (60.97, 810.15)	78.39 (20.38, 201.00)
Precision for interaction index (Region and month)	6.84 (6.25, 7.37)	26.23 (15.80, 42.57)

* Statistically significant at $5\%$ level, as indicated by the $95\%$ CI excluding 1

**Fig. 5:**
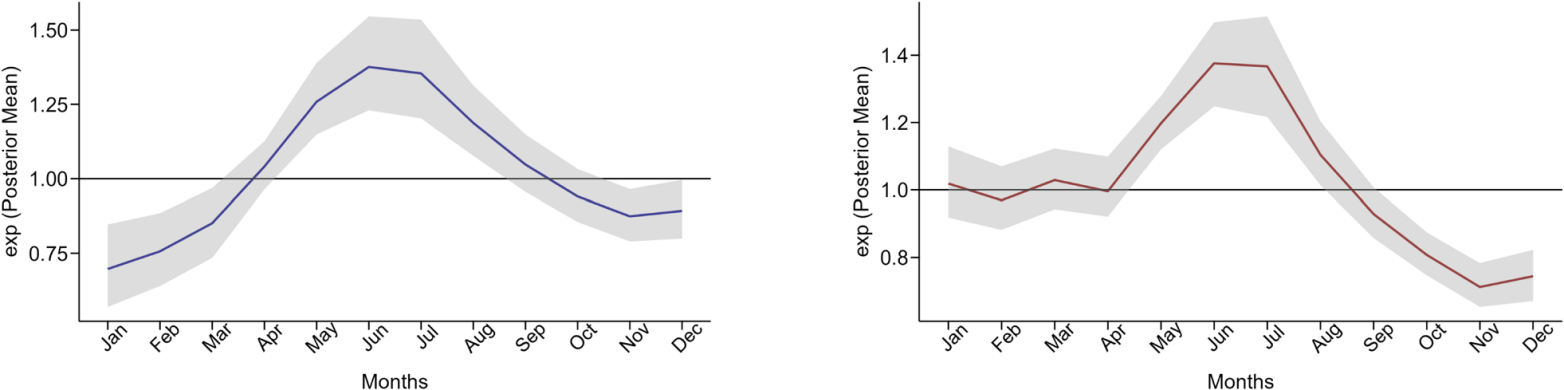
Exponential posterior mean for temporal trend with its credible intervals for children under five years (left) and for individuals aged five years and above (right)

We assessed the posterior temporal trend effect for malaria relative risk for the CAR component for the two age groups (children under five years of age and individuals aged five years and above); see Figure 5. The general trend reveals an increased risk of malaria from February to July followed by a decrease from September to November, and then start to increase again from December. Comparing these trends with the time series plots of monthly malaria cases (Figure 1b and c), we find indications that the random walk time model effectively captures the observed temporal patterns in the malaria incidence.

**Fig. 6:**
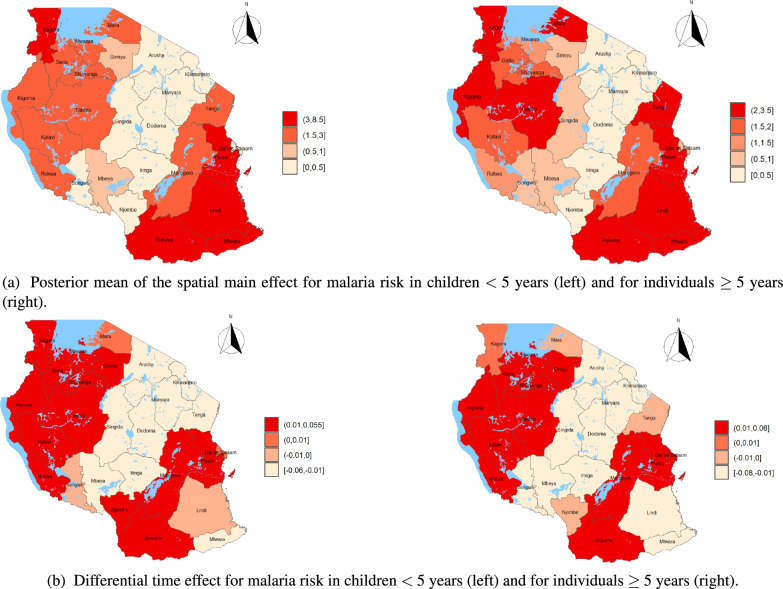
Posterior mean of the spatial main effect $\zeta _i = \exp (u_i+v_i)$ (a) and the differential time effect $\delta _i$ (b) for malaria risk in Tanzania mainland regions for children $< 5$ years (left) and for individuals $\ge 5$ years (right), respectively. These maps illustrate spatial heterogeneity in malaria risk across different regions. Areas are shaded in varying intensities to represent the range of effects, with red colors indicating regions with relative malaria risk.

**Fig. 7:**
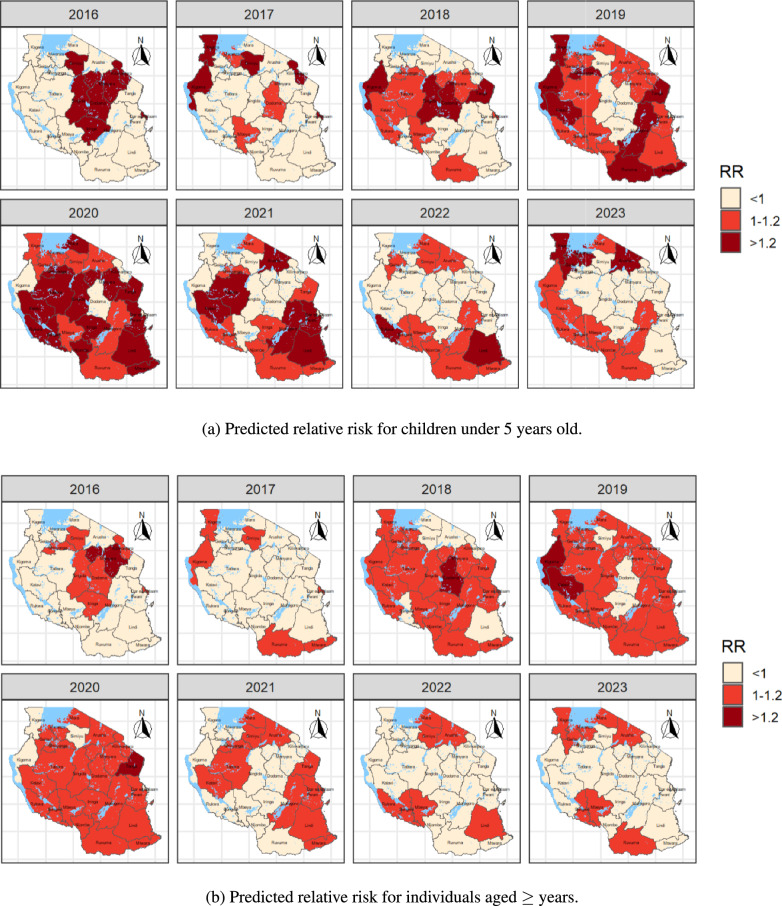
Yearly averaged predicted relative risk of the spatio-temporal interaction $\delta _{it}$ (nonspatially or temporally structured interaction) across the 26 Tanzania mainland regions

The maps of the posterior mean of the spatial effect, $\zeta _i = \exp (u_i + v_i)$, and the differential time effect, $\delta _i$, for malaria risk across Tanzania mainland regions are presented in Figure 6 for the two demographic groups: children under five years of age and individuals aged five years and above. Figure 6a focuses on the spatial effects, which capture the underlying geographic variation in malaria risk. These maps reveal that regions in the south (Ruvuma, Mtwara, and Lindi), the Lake Victoria zone (Kagera, Geita, Mwanza, Shinyanga, and Mara), the west (Kigoma, Katavi, Rukwa, and Tabora), parts of the central zone (Morogoro), and the eastern coastline (Pwani, Dar es Salaam, and Tanga) consistently show increased spatial effects, signaling a higher baseline malaria risk. In contrast, lower spatial effects are observed in the central, southern highlands, and northern highlands regions, such as Dodoma, Singida, Mbeya, Songwe, Njombe, Manyara, Arusha, and Kilimanjaro, suggesting a reduced underlying risk of malaria in these regions. Complementing the spatial patterns, the differential time effects shown in Figure 6b illustrate the posterior probabilities used to assess whether region-specific malaria trends significantly differ from the national average trend. A value of $\delta _i > 0$ (shaded in red) indicates that the trend in a particular region is steeper than the national mean, suggesting that malaria risk is increasing more rapidly in that region. Conversely, a value of $\delta _i < 0$ (shown in lighter shades) suggests a less steep trend, indicating that malaria risk is decreasing or increasing more slowly than the national average. These temporal dynamics closely mirror the spatial patterns: regions with high spatial effects, particularly those in the east, south, and Lake Zone, also tend to exhibit above-average increases in malaria risk over time. Conversely, regions in the north, central areas, and southern highlands not only show lower spatial effects but also demonstrate flatter or decreasing temporal trends. Note that, the posterior mean of the spatio-temporal interaction, $\delta _{it}$, for malaria cases in Tanzania mainland for both age groups is presented in Appendix A (Figure 7).

## Discussion

Model-based malaria surveillance that integrates weather variables is increasingly recognized as a crucial strategy for mitigating the impact of climatic variability on malaria outbreaks [12, 42]. In this study, we applied the Standardized Incidence Ratio (SIR) to assess regional malaria risk across Tanzania mainland and employed a Bayesian spatio-temporal modeling approach to analyze the influence of climatic factors and disease interventions on malaria cases. Our findings revealed a significant reduction in malaria risk during the study period (2016–2023): an $11.0\%$ decrease among children under five years of age and a $10.0\%$ decrease among individuals aged five years and above. This decline may be partly attributed to disruptions caused by the COVID-19 pandemic, which affected healthcare service delivery and likely contributed to underreporting and decreased healthcare-seeking behavior. As observed in Figure 1, malaria cases in both age groups exhibited an increasing trend from 2016 to 2019, followed by a notable decline beginning in 2020. Furthermore, the distribution of long-lasting insecticidal nets (LLINs) significantly reduced malaria risk, with a $1\%$ increase in LLIN coverage leading to a $1.2\%$ decrease in risk among children under five years and a $7.0\%$ decrease among individuals aged five years and above. Several climatic factors were also identified as significant drivers of malaria risk across both age groups, including relative humidity, minimum temperature, wind speed, and vegetation indices. Maximum and minimum temperatures consistently emerged as key predictors, with specific time lags influencing malaria incidence differently across age groups. These findings provide crucial insights for the Tanzania National Malaria Control Programme (NMCP) by highlighting the importance of integrating climatic factors into surveillance systems to predict and manage malaria risk. Moreover, the results can support localized efforts by guiding county health departments in tailoring malaria control interventions to regional conditions and population dynamics.

Temperature plays a crucial role in driving malaria transmission by influencing both the survival and development of mosquito vectors and the malaria parasite. Higher temperatures accelerate the mosquito blood-feeding cycle, which is essential for the transmission of malaria [4, 43, 44]. Our study found that the average minimum temperatures during the month of reported malaria cases were associated with an increased risk of malaria for both age groups (Table 4). Specifically, increased minimum temperatures one month prior significantly increased the risk malaria for individuals aged five years and above, while increased minimum temperatures three months prior had a similar effect on children under five years of age. In contrast, for maximum temperature a different scenario was observed: increased maximum temperatures during the month of reported malaria cases had a significant negative impact on malaria risk for both children under five years of age and individuals aged five years and above. Furthermore, maximum temperatures one and two months prior to the reported malaria cases also exhibited a significant negative effect on malaria risk for individuals aged five years and above and for children under five years of age, respectively. These results align with previous studies. For instance, research in Lower Moshi, Tanzania, found that malaria test positivity rates were positively correlated with average monthly minimum temperatures and negatively correlated with average monthly maximum temperatures [19]. Other studies have shown that temperatures below 27$^\circ$C are associated with higher malaria incidence rates [45, 46], consistent with our study’s finding that the highest recorded minimum temperature was 26.30$^\circ$C. Conversely, temperatures above 30$^\circ$C were linked to reduced malaria incidence [47, 48], which aligns with our observation of an average maximum temperature of 30.17$^\circ$C.

Precipitation and relative humidity are key determinants in the dynamics of malaria transmission. The intensity and duration of precipitation are crucial in creating aquatic habitats conducive to mosquito breeding. While the water pools need to persist long enough for mosquito larval development, excessive precipitation can result in high immature mosquito mortality [49]. Given the varying breeding preferences of *Anopheles* mosquitoes and the influence of other environmental factors, the impact of precipitation on malaria incidence has produced inconsistent findings, with studies reporting positive [50–52], negative [53], or non-significant [54] correlations. In this study, we observed that precipitation did not significantly affect the risk of malaria. Moreover, our study identified a non-significant association between current relative humidity and an increased risk of malaria cases across both age groups. However, at a lag of two months, relative humidity was associated with an increased malaria risk for children under five years of age (Table 4). This observation is consistent with existing literature, which highlights a positive association between malaria transmission and relative humidity [55, 56]. However, other studies have shown that relative humidity and malaria incidence have a negative correlation [57, 58], while some show that they have no significant correlation [19].

We found that incorporation of Normalized Difference Vegetation Index (NDVI), which serves as a surrogate for vegetation response to rainfall, showed that the risk of malaria was higher in areas with dense vegetation compared to areas with sparse or no vegetation in both age groups. Dense vegetation likely provides favorable conditions for mosquito breeding and survival, such as increased humidity and more breeding sites, which in turn raises the risk of malaria transmission. Similar observations have been reported in studies conducted in Uganda [59], Mozambique [53], Nigeria [60], and Ivory Coast [61], where regions with higher NDVI values, indicative of dense vegetation, were associated with increased malaria risk.

Vector control interventions are pivotal in the fight against malaria, significantly reducing the incidence and prevalence of this disease. Among these interventions, long-lasting insecticidal nets (LLINs) are particularly effective, providing a physical barrier against mosquito bites and delivering insecticides that kill mosquitoes upon contact. The widespread use of LLINs has been a cornerstone in malaria control strategies across sub-Saharan Africa, including Tanzania. Our study has shown that usage of LLIN was associated with a reduction in malaria risk across both age groups (Table 4), with a more substantial impact observed in individuals aged five years and above. This disparity could be due to several factors, including differences in exposure patterns, immunity levels, and adherence to LLIN use. Younger children, while benefiting from LLINs, may still be vulnerable due to their weaker immune systems and greater exposure during peak mosquito activity times. These findings have significant implications for malaria control strategies in Tanzania. The evident protective effect of LLINs, particularly in older individuals, underscores the importance of maintaining high coverage and consistent use of LLINs. For children under five, additional interventions may be necessary to complement LLIN usage, such as targeted indoor residual spraying (IRS), community health education, and improving access to prompt malaria diagnosis and treatment.

The spatio-temporal model, which was divided into spatial and temporal effects, each with structured and unstructured heterogeneity of malaria cases, revealed interesting patterns in the non-parametric dynamics. The structured temporal effect showed fluctuations over the study period, with malaria cases peaking between January and July, following the main rainy seasons. The structured spatial effect varied across different regions, with the eastern, western, southern, and lake zones experiencing higher effects compared to the central and northern zones. These findings align with research by Gosoniu et al. [62], based on data from the 2007/2008 Tanzania HIV/AIDS and Malaria Indicator Survey (THMIS), which predicted high malaria prevalence in regions around Lake Victoria (Kagera, Mara, and Shinyanga) and the southern part of the country (Pwani, Lindi, Mtwara, and Ruvuma provinces). This indicates that these zones experienced more significant variations in malaria cases and had higher incidence rates.

The spatio-temporal trend model employed in this study stands out for several reasons, making it a valuable tool for shaping policy decisions related to malaria prevention and control. By integrating spatial and temporal data, the model can accurately predict areas and periods of high risk, facilitating targeted interventions and resource allocation. Another strength lies in the adaptability of the model, allowing it to be applied in different locations with similar data availability, adding a practical dimension to its utility. The regional-level trends identified in the study provide valuable insights for regional health offices to assess the effectiveness of malaria prevention efforts. Moreover, the study takes into account the impact of climate and malaria transmission interventions. It is however crucial to note that the model is most suitable for the study area during the specified period and cannot be universally applied to other locations or time periods without re-estimating all model parameters. Despite this, some transmission dynamics parameters are expected to be similar in analogous malaria settings, particularly in moderate to high transmission environments. It is worth mentioning that our models did not consider other potential factors influencing malaria transmission beyond climate and LLINs (as an intervention). Nevertheless, these un-utilized factors have been partially taken into account by the random effects incorporated into the model.

The model demonstrates its potential for long-term planning and resource allocation by accurately forecasting trends in malaria cases. In the predicted trends for 2023, the forecasted cases closely matched the observed data (Figure 4). Although it is not designed as a traditional early warning system (which typically predicts outbreaks within a two-week window), the model highlights key climatic factors that can be used to refine predictions of malaria risk over time and across different regions. Additionally, the malaria risk maps produced by the model as regionally aggregated estimates, are valuable tools for local health departments, helping to guide timely interventions such as vector control measures and resource allocation. However, it is important to note that using only malaria case data may cause delays in outbreak predictions due to the lag between mosquito activity and confirmed malaria cases, which results from the disease’s incubation period. To improve prediction timeliness, incorporating entomological data-such as mosquito population monitoring-could provide earlier warnings of transmission risks. Mosquito surveillance provides a direct and real-time indicator of vector presence, which could complement malaria case data and enhance the model’s effectiveness in forecasting outbreaks.

## Conclusion

In this study, we utilized the Standardized Incidence Ratio (SIR) to evaluate the regional distribution of malaria risk across Tanzania mainland regions and applied Bayesian spatio-temporal models to examine the influence of climatic factors and disease interventions on malaria incidence in two groups: children under five years of age and individuals aged five years and above. Our analysis revealed a clear temporal pattern, with malaria cases peaking from November to July, coinciding with the main rainy seasons. The risk of malaria was clustered in specific regions, with the eastern, western, southern, and lake zones experiencing higher effects compared to the central and northern zones. The study showed a notable reduction in malaria risk during the study period, and the use of Long-lasting insecticidal nets (LLINs) were found to significantly reduce the risk of malaria in both age groups. Climatic factors, including relative humidity, minimum temperature, and vegetation indices, were associated with an increased risk of malaria, with specific month lags amplifying the influence of these factors differently across age groups. The malaria risk maps produced by the model are valuable tools for local health departments. They can guide timely interventions, such as vector control measures and resource allocation, to mitigate malaria transmission effectively. By understanding and addressing the climatic and environmental predictors of malaria, health authorities can better plan and implement targeted strategies to reduce malaria incidence.

## Data Availability

The malaria cases, interventions and population data are available from the Tanzania National Health Portal (https://hmisportal.moh.go.tz/hmisportal/#/home). Climatic/environmental data are available online from the sites described in the manuscript.

## Appendix A

See Fig. 7
